# Subclinical Influenza Virus A Infections in Pigs Exhibited at Agricultural Fairs, Ohio, USA, 2009–2011

**DOI:** 10.3201/eid1812.121116

**Published:** 2012-12

**Authors:** Andrew S. Bowman, Jacqueline M. Nolting, Sarah W. Nelson, Richard D. Slemons

**Affiliations:** The Ohio State University, Columbus, Ohio, USA

**Keywords:** Influenza virus A, swine, subclinical, infection, livestock, exhibits, viruses, influenza, Ohio, United States, agriculture, human, zoonoses, pigs

## Abstract

Agricultural fairs are associated with bidirectional, interspecies transmission of influenza virus A between humans and pigs. We examined pigs exhibited at agricultural fairs in Ohio during 2009–2011 for signs of influenza-like illness and collected nasal swab specimens from a representative subset of these animals. Influenza virus A was recovered from pigs at 12/53 (22.6%) fairs during the 3-year sampling period. Pigs at 10/12 (83.3%) fairs from which influenza virus A was recovered did not show signs of influenza-like illness. Hemagglutinin, neuraminidase, and matrix gene combinations of the isolates were consistent with influenza virus A concurrently circulating among swine herds in the United States. Subclinical influenza virus A infections in pigs at agricultural fairs may pose a risk to human health and create challenges for passive surveillance programs for influenza virus A in swine herds.

## Abstract 2

Close contact between pigs and humans could result in zoonotic transmission.

Awareness of bidirectional zoonotic transmission of influenza virus A between pigs and humans was heightened by the emergence of the influenza A(H1N1)pdm09 virus, which resulted in an influenza pandemic among humans starting in 2009. Interspecies transmission of influenza virus A is believed to be a principal mechanism contributing to the emergence of novel influenza virus A strains that pose a threat to human and swine health (*1*,*2*). Pig respiratory tracts have receptors for swine-, human-, and avian-origin influenza virus A, which facilitates genomic reassortment among viruses from multiple host species. As a result, swine have been identified as mixing vessels for influenza virus A and a source of emergence for novel viruses (*3*).

For >60 years after its identification as a swine pathogen, influenza virus A circulating among North American swine was predominantly the H1N1 subtype (*4*). In 1998, triple-reassortant influenza virus A (H3N2), containing genes originating from swine-, human-, and avian-origin influenza virus A, was identified among swine in the United States (*5*). This lineage quickly became established among North American swine (*6*), and the 6 gene segments coding for internal proteins, including the matrix (M) gene, subsequently served as a common backbone for many new reassortant viruses appearing among pigs (*7*). Various subtype H1N1, H1N2, and H3N2 influenza virus A lineages continue to cocirculate and evolve among North American swine (*6*–*9*).

Swine are a source of novel and existing influenza virus A strains that infect humans (*10*–*13*). These strains pose a pandemic threat if they become capable of being transmitted efficiently from person to person and if limited protective immunity exists in the human population. Bidirectional zoonotic transmission of influenza virus A strains usually involves close contact between humans and swine. The United States has 3 major swine–human interfaces: commercial swine production, abattoirs, and agricultural fairs. Agricultural fairs are unique because they facilitate prolonged commingling of pigs from numerous sources raised under varied management programs with millions of persons who have widely disparate histories of exposure to various influenza viruses. This situation creates an environment conducive to zoonotic transmission of influenza virus A.

More persons come in contact with live swine at agricultural fairs than in any other setting in the United States, and several human cases of influenza A have been linked to swine exposure occurring at fairs. In 1988, a woman died of infection with a variant influenza virus A (H1N1) that she acquired while attending a Wisconsin fair where numerous pigs showed signs of influenza-like illness (ILI); a follow-up investigation identified more human infections (*14*). In Ohio, human infections with variant influenza virus A after exposure to pigs with ILI were detected at the 1988 Ohio State Fair, 2 weeks before the Wisconsin case was reported (R.D. Slemons, unpub. data), and more recently at the 2007 Huron County Fair (*15*).

Because of dynamic human and swine populations at fairs and the number of human influenza A cases associated with swine exposure that occurs at fairs (*13*–*15*), we hypothesized that influenza virus A infections in swine occur undetected at agricultural fairs. This study was initiated after the emergence of influenza A(H1N1)pdm09 to actively monitor the antigenic and genomic properties of influenza virus A among pigs at agricultural fairs in Ohio, with a goal of protecting the health of swine and the public.

## Materials and Methods

### Study Sites and Samples

During each year of this 3-year study, 2009–2011, agricultural fairs in Ohio were strategically recruited to participate on the basis of the size of the county’s commercial swine industry, the number of 4-H/FFA swine exhibitors, the number of pigs previously exhibited, or the geographic proximity to study sites used for influenza virus A surveillance in wild birds. Selection criteria were chosen to provide a diverse representation of Ohio’s exhibition swine and the influenza virus A strains they might harbor. Before visiting the fair, the study team provided the leaders of each participating agricultural fair with an educational fact sheet on swine influenza.

The agricultural fair season in Ohio begins in June and continues into October; the fairs participating in this study occurred throughout the fair season (Figure). Fairs were visited at the end of the swine exhibition period, at which time pigs were visually examined for signs of ILI, and nasal swab specimens were collected from 20 selected pigs representing all areas of the exhibit, without consideration for individual pig health status (healthy or ill). Each nasal swab was placed in an individual vial containing brain–heart infusion broth supplemented with penicillin and streptomycin (*16*). The samples were frozen at −70°C until testing was initiated. The Institutional Animal Care and Use Committee of The Ohio State University approved protocol no. 2009A0134 for the use of animals in this study.

**Figure:**
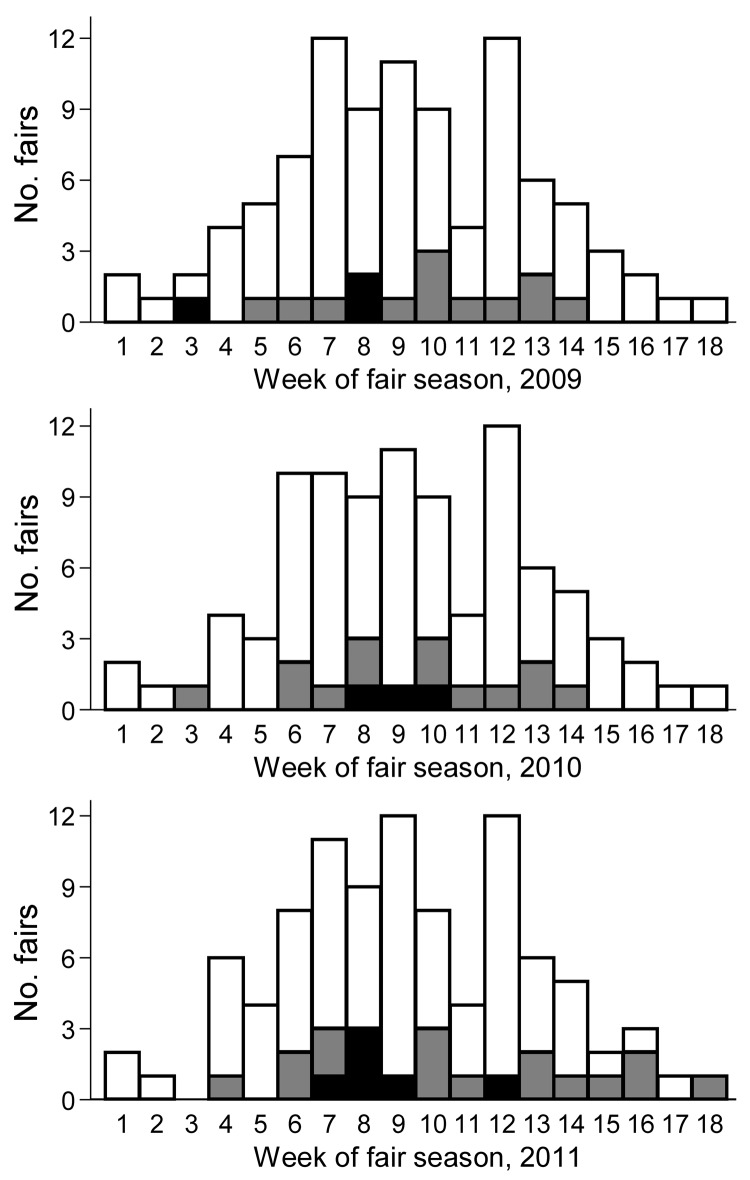
Frequency distribution of agricultural fairs, by week of the state fair season, Ohio, June–October 2009–2011. Black bar sections, fairs with pigs positive for influenza virus A; gray bar sections, fairs with no pigs positive for influenza virus A; white bar sections, fairs not enrolled in this study.

### Virus Isolation from Swine Nasal Swab Specimens

Samples were thawed and treated with amphotericin B, gentamicin sulfate, and kanamycin sulfate (*17*); they then underwent centrifugation at 1,200 × *g* for 30 min at 4°C. The brain–heart infusion broth supernatant was added to 24-well plates containing monolayers of Madin-Darby canine kidney (MDCK; catalog no. 84121903, Sigma-Aldrich Co., St. Louis, MO, USA), adapted to and maintained in serum-free medium (A.S. Bowman et al., unpub. data). MDCK monolayers were examied for cytopathic effect (CPE) daily for 3 days after the supernatant was added, at which time the cell culture supernatant was tested for hemagglutination activity by using 0.5% turkey erythrocytes (*18*). All hemagglutinating agents in cell culture supernatant and all MDCK cell cultures showing CPE were tested for the presence of influenza virus A by using Flu DETECT (Synbiotics Corporation, Kansas City, MO, USA). Each cell culture supernatant that had positive test results with Flu DETECT was identified as an influenza A viral isolate. Influenza virus A isolates were further characterized by using real-time reverse transcription PCR (rRT-PCR) assays.

### RNA Extraction and rRT-PCR

RNA was extracted from original samples and influenza virus A isolates by using the PrepEase RNA Spin Kit (Affymetrix, Inc. Cleveland, OH, USA) according to the manufacturer’s instructions. Pan–influenza virus A rRT-PCR (*19*,*20*) was used to screen all original samples for influenza virus A. Hemagglutinin (HA) and neuraminidase (NA) subtypes of the influenza isolates were determined by using rRT-PCR assays specific for swine-origin influenza A virus H1 and H3 HA genes and N1 and N2 NA genes by using either a previously published protocol (*21*) modified to laboratory conditions (A.S. Bowman et al., unpub. data) or a commercially available swine influenza virus subtyping assay (Applied Biosystems, Foster City, CA, USA).

The M gene of the influenza A virus isolates was further characterized by differentiating between the North American swine triple-reassortant and the influenza A(H1N1)pdm09 virus M genes by using an rRT-PCR protocol (*22*) adapted to laboratory conditions. The reactions were carried out by using the QuantiFast Multiplex RT-PCR +R Kit (QIAGEN, Valencia, CA, USA) in a 20-μL reaction mixture containing 10 μL 2× quantitative RT-PCR master mix, 7.5 pmol of forward primer, 2.5 pmol of each reverse primer, 0.125 μmol/L EA minor groove binder probe, 0.0625 μmol/L of each NA minor groove binder probe, 0.4 μL 50× ROX reference dye, 0.2 μL of reverse transcription product, and 5 μL of extracted RNA. The reactions were performed on an Mx3000P QPCR System (Agilent Technologies, Inc., Santa Clara, CA, USA) under these thermocycling conditions: 50°C for 20 min, then 95°C for 5 min, followed by 50 cycles of 97°C for 2 s and 60°C for 40 s. Cycle threshold values were calculated for each sample automatically by the QPCR System’s software by using the background-based method. Samples with cycle threshold <40 were considered positive.

## Results

Fifty-three fair events were included in this study: 15 fairs during 2009, 16 fairs during 2010, and 22 fairs during 2011(Figure). Influenza virus A was recovered from >1 pigs at 12/53 (22.6%) fair events (Table 1). Results of the pan–influenza virus A rRT-PCR performed on original samples and virus isolation were completely concordant. Pigs with signs of ILI were observed and sampled at 2/53 (3.7%) fair events, and influenza A virus isolates were recovered from pigs at both fairs; pigs without signs of ILI but with positive test results for influenza A virus were found at 10/53 (18.9%) fair events. Therefore, pigs at 10/12 (83.3%) fairs at which pigs had influenza virus A infection did not exhibit signs of ILI.

**Table 1:** Clinical signs of ILI and influenza virus A recovery from pigs at agricultural fairs, Ohio, USA, 2009–2011*

Year	No. participating fairs	No. fairs with pigs with ILI	No. (%) fairs with >1 pig testing positive for influenza virus A	No. (%) fairs with subclinical influenza virus A infection in pigs
2009	15	0	3 (20.0)	3 (20.0)
2010	16	1	3 (18.8)	2 (12.5)
2011	22	1	6 (27.3)	5 (22.7)

*Influenza A virus was recovered from pigs at both fairs where there were pigs with ILI. ILI, influenza-like illness.

A total of 1,073 pigs were tested during the 3-year study; influenza virus A was recovered from 155 (14.4%). The frequencies of virus isolation by year were 40/299 (13.4%) during 2009, 34/315 (10.8%) during 2010, and 81/459 (17.7%) during 2011. For the 12 fairs with >1 pigs testing positive for influenza A virus, the average frequency of virus isolation from nasal swab specimens was 62.9% (range 5%–100%; Table 2).

**Table 2:** Frequency of influenza virus A isolation from individual pigs exhibited at agricultural fairs with >1 pig testing positive for influenza virus A, Ohio, USA, 2009–2011*

Fair	No. pigs positive/no. tested (%)		
	2009	2010	2011
A	18/20 (80)	0/20	0/20
B	10/20 (50)	0/20	20/20 (100)
C	19/20 (90)	15/20 (75)	20/20 (100)†
D	0/20	1/20 (5)†	19/20 (95)
E	0/20	18/20 (90)	0/20
F	0/20	0/20	3/20 (15)
G		0/20	3/20 (15)
H			16/40 (40)

*Fair G did not participate in 2009; fair H did not participate in 2009 or 2010. †Fairs where there were pigs with influenza-like illness.

Influenza virus A subtypes recovered were H1N2 and H3N2 during 2009, H3N2 during 2010, and H1N2 and H3N2 during 2011 (Table 3). The North American swine triple-reassortant M gene was found in all isolates recovered during 2009 and 2010, whereas the M gene from the influenza A(H1N1)pdm09 virus was found in all of the 2011 H3N2 and H1N2 isolates (Table 3).

**Table 3:** Characterization of HA, NA, and M gene segments of influenza virus A from agricultural fairs with >1 pig testing positive for influenza virus A, Ohio, USA, 2009–2011*

Fair	Year		
	2009	2010	2011
A	**H1N2**	Negative	Negative
B	**H3N2**	Negative	H3N2
C	**H3N2**	**H3N2**	H1N2, H3N2
D	Negative	**H3N2**	H1N2, H3N2
E	Negative	**H3N2**	Negative
F	Negative	Negative	H3N2
G		Negative	H3N2
H			H1N2, H3N2

*Fair G did not participate in 2009; fair H did not participate in 2009 or 2010. **Boldface** indicates North American triple-reassortant swine-origin influenza A virus M gene segment; underlining indicates influenza A(H1N1)pdm09 virus M gene segment. HA, hemagglutinin; NA, neuraminidase; M, matrix.

## Discussion

Our findings highlight the limitations of relying on visual examination for ILI to identify pigs infected with influenza virus A at agricultural fairs. Subclinical influenza virus A infections predominated among the pigs we tested, with subclinical infections detected among pigs at 10/53 (18.9%) participating fairs during 2009–2011. These findings may explain the frequency of variant influenza virus A infections among humans who have only been exposed to apparently healthy swine at fairs.

Agricultural fairs are often the face of agriculture to the general public. The International Association of Fairs and Expositions estimates annual attendance at fairs in North America to be 150 million persons (The Association, pers. comm.). Agricultural fairs have been occurring in the United States since 1811 (*23*) and are special community events with a strong tradition and history of celebrating agricultural heritage and achievement (*24*). As the agricultural workforce has decreased to <2% of the US population (*25*), fairs have added educational programs to showcase advancements in food production systems in an effort to maintain attendance (*26*) and meet societal needs. These much-needed educational efforts often provide an opportunity for attendees to have direct contact with all facets of agriculture, including pork production. Many of these persons would not otherwise have any exposure to swine and the pathogens they harbor, so their close contact with pigs at fairs may play multiple roles in the transmission of influenza A viruses: they may pass human-origin influenza virus A to swine, leading to novel reassortant viruses; they may serve as early sentinels by becoming infected first with a novel swine-origin influenza A virus; or they may disseminate a novel swine-origin influenza virus A in their local communities (*27*).

The long duration of many agricultural fairs (3–10 days) is distinctly different than other swine concentration points or commingling events (i.e., abattoirs, markets, auctions, or shows), where interactions are limited to hours. In addition to their long duration, agricultural fairs also enable the comingling of pigs from multiple locations and various production systems (backyard to intensive commercial) at 1 site. Exhibition swine are commonly a unique population of noncommercial swine, reared by the use of management practices that differ greatly from standard commercial swine production practices (*28*). These pigs likely have varying levels of immunity to influenza virus A and may bring a variety of influenza virus A strains with them to the fair, where the viruses can spread to other pigs, possibly reassort, and potentially transmit to humans.

Swine-to-human transmission of influenza virus A has been sporadically reported worldwide (*11*), but the true incidence of this transmission is unknown. The Centers for Disease Control and Prevention reported that 36 humans were infected with variant influenza virus A in the United States during December 2005–April 2012 (*29*). Of these cases, 15 occurred after July 2011, and 6 cases, all involving infection with influenza A (H3N2) viruses containing the M gene from the influenza A(H1N1)pdm09 virus (H3N2v), were associated with exposure to swine at agricultural fairs. However, none of the implicated fairs reported signs of ILI in the pigs, and influenza virus A could not be isolated from the pigs that were suspected to be the sources because of delays and lack of the availability of the pigs. Nonetheless, it is possible that subclinical influenza infections in pigs at these swine–human interfaces played a key role in zoonotic infections.

The increased swine–human exposure occurring at agricultural fairs may also facilitate human-origin influenza A virus transmission to swine. The earliest reports of introductions of the influenza A(H1N1)pdm09 virus into the US swine herd occurred at the state fairs in Minnesota and South Dakota (*30*,*31*). Human-to-swine transmission is credited as a primary source of the genetic diversity seen in currently circulating swine influenza virus strains (*32*–*34*). Human-to-swine transmission of influenza virus A can be economically devastating for the pork industry because of decreased domestic sales, restrictions imposed by export partners, and production losses due to disease. Agricultural fairs may provide a conduit to introduce human-origin influenza virus A into the US swine herd.

No human cases of variant influenza A associated with any of the agricultural fairs included in this study were reported, even though influenza A (H3N2) viruses containing the M gene from the influenza A(H1N1)pdm09 virus were recovered from pigs at 6 of the participating fairs in 2011. However, the number of confirmed H3N2v cases dramatically increased during the summer of 2012, with most cases epidemiologically linked to swine exposure occurring at agricultural fairs (*35*,*36*).

The HA, NA, and M gene combinations of the influenza virus A isolates recovered from 155/1,073 (14.4%) sampled pigs were consistent with influenza virus A concurrently circulating in the US swine population (*37*,*38*). The high frequency of virus isolation from the pigs at the 12 fairs at which influenza virus A was found is likely due to sample collection occurring at the end of the exhibition period, ≈5–7 days after arrival, which probably coincided with peak viral shedding in the swine population.

A limitation of the study is that extrapolating the findings to other Ohio fairs and fairs in other states may not be possible because of the selection bias and inherent variability among agricultural fairs. Although the fairs where influenza virus A was recovered were diverse regarding the predetermined selection criteria (data not shown), the participating fairs were included in the study because they were ranked relatively high among Ohio fairs within >1 selection category. Expanded surveillance efforts for agricultural fairs are underway to more accurately estimate the true prevalence of influenza virus A infections among swine at agricultural fairs in Ohio. Recognized risk factors and accurate prevalence estimates are needed to lay the foundation for studies investigating potential interventions to decrease the probability of swine-to-human and human-to-swine transmission of influenza virus A at agricultural fairs.

The subclinical influenza virus A infections identified in this study would not be detected by the current national swine influenza virus surveillance program (*39*), which is passive and focuses on swine showing signs of ILI and on reacting to reports of variant influenza A cases in humans (*39*). Thus, subclinical influenza virus A infections among pigs are likely underreported. This passive surveillance strategy does not adequately describe the breadth of influenza virus A circulating in swine because it does not identify less virulent strains of influenza virus A (*40*) and does not collect metadata on host, environmental, and agent factors that affect severity of illness. Therefore, to accurately capture the risk influenza virus A in swine populations presents to swine and public health, surveillance efforts should include healthy and clinically ill pigs.

Reducing bidirectional zoonotic transmission of influenza virus A between pigs and humans is crucial to agriculture and biomedicial science. Unfortunately, little scientific evidence exists on which to base changes in policies and management practices to reduce the risk for interspecies transmission of influenza virus A between pigs and humans. This investigation highlights the need for additional studies to quantify the risk for interspecies influenza A virus transmission at fairs and to evaluate interventions to mitigate the risk.

Potential strategies to mitigate the risk for intra- and interspecies transmission of influenza virus A at fairs on the swine side of the human-swine interface include shortening the swine exhibition period, preventing interfair movement of pigs, and vaccinating exhibition swine for appropriate influenza A viruses. Recommendations have previously been made for mitigating risk on the human side of the human–swine interface (www.cdc.gov/flu/swineflu). Expanded risk assessments at agricultural fairs will provide animal and public health officials with scientific data that will enable them to make appropriate decisions to protect animal and public health while still furthering appreciation and understanding of agriculture and ensuring our future food security.
